# Synthesis, Characterization, and Histological Evaluation of Chitosan-Ruta Graveolens Essential Oil Films

**DOI:** 10.3390/molecules25071688

**Published:** 2020-04-07

**Authors:** Carlos David Grande Tovar, Jorge Iván Castro, Carlos Humberto Valencia Llano, Diana Paola Navia Porras, Johannes Delgado Ospina, Mayra Eliana Valencia Zapata, José Herminsul Mina Hernandez, Manuel N. Chaur

**Affiliations:** 1 Grupo de Investigación de Fotoquímica y Fotobiología, Universidad del Atlántico, Carrera 30 Número 8-49, Puerto Colombia 081008, Colombia; carlosgrande@mail.uniatlantico.edu.co; 2 Grupo de Investigación SIMERQO, Departamento de Química, Universidad del Valle, Calle 13 No. 100-00, Cali 76001, Colombia; jorge.castro@correounivalle.edu.co; 3 Escuela de Odontología, Grupo biomateriales dentales, Universidad del Valle, Calle 4B # 36-00, Cali 76001, Colombia; carlos.humberto.valencia@correounivalle.edu.co; 4 Grupo de Investigación Biotecnología, Facultad de Ingeniería, Universidad de San Buenaventura Cali, Carrera 122 # 6-65, Cali 76001, Colombia; dpnavia@usbcali.edu.co (D.P.N.P.); jdelgado1@usbcali.edu.co (J.D.O.); 5 Escuela de Ingeniería de Materiales, Facultad de Ingeniería, Universidad del Valle, Calle 13 No. 100-00, Santiago de Cali 760032, Colombia; valencia.mayra@correounivalle.edu.co; 6 Centro de Excelencia en Nuevos Materiales (CENM), Universidad del Valle, Calle 13 No. 100-00, Santiago de Cali 760032, Colombia

**Keywords:** biocompatibility, chitosan films, *Ruta graveolens* essential oil, scaffolds

## Abstract

The development of new biocompatible materials for application in the replacement of deteriorated tissues (due to accidents and diseases) has gained a lot of attention due to the high demand around the world. Tissue engineering offers multiple options from biocompatible materials with easy resorption. Chitosan (CS) is a biopolymer derived from chitin, the second most abundant polysaccharide in nature, which has been highly used for cell regeneration applications. In this work, CS films and *Ruta graveolens* essential oil (RGEO) were incorporated to obtain porous and resorbable materials, which did not generate allergic reactions. An oil-free formulation (F1: CS) and three different formulations containing *R. graveolens* essential oil were prepared (F2: CS-RGEO 0.5%; F3: CS+RGEO 1.0%; and F4: CS+RGEO 1.5%) to evaluate the effect of the RGEO incorporation in the mechanical and thermal stability of the films. Infrared spectroscopy (FTIR) analyses demonstrated the presence of RGEO. In contrast, X-ray diffraction (XRD) and differential scanning calorimetry (DSC) analysis showed that the crystalline structure and percentage of CS were slightly affected by the RGEO incorporation. Interesting saturation phenomena were observed for mechanical and water permeability tests when RGEO was incorporated at higher than 0.5% (*v/v*). The results of subdermal implantation after 30 days in Wistar rats showed that increasing the amount of RGEO resulted in greater resorption of the material, but also more significant inflammation of the tissue surrounding the materials. On the other hand, the thermal analysis showed that the RGEO incorporation almost did not affect thermal degradation. However, mechanical properties demonstrated an understandable loss of tensile strength and Young’s modulus for F3 and F4. However, given the volatility of the RGEO, it was possible to generate a slightly porous structure, as can be seen in the microstructure analysis of the surface and the cross-section of the films. The cytotoxicity analysis of the CS+RGEO compositions by the hemolysis technique agreed with in vivo results of the low toxicity observed. All these results demonstrate that films including crude essential oil have great application potential in the biomedical field.

## 1. Introduction

Since the early adoption of the term “tissue engineering”, several studies have been published, demonstrating three-dimensional scaffolds development with the ability to add cells and support their proliferation in a wide range of applications [1]. On the other hand, of significant importance in the design of scaffolds are the final architecture, excellent mechanical properties, and biocompatibility, in addition to cell adhesion, proliferation, and differentiation abilities [2]. Chemical composition and structural modifications of the materials selected for scaffold design are the ultimate aspects to determine the success in tissue-engineering applications [3,4].

Natural or synthetic polymer-based scaffolds have advantages and disadvantages in tissue engineering applications. Synthetic polymers can be produced under controlled conditions, and their mechanical and physical properties predicted [5]. Synthetic polymers are also highly used since they are cheap, and their properties (porosity, mechanical, and thermal resistance) can be tailored design. However, low degradation rates and low cell adhesion and interaction compared to natural counterparts are the main disadvantages.

The use of natural scaffolds in tissue engineering includes bone, cartilage, ligament, and skin, among others [6]. There are several advantages of using natural polymers-based scaffolds. For example, skin injuries using biomaterials exhibited better vascularization, better integration, and lower health risks than those from autologous skin grafts and split-skin grafts, becoming an option adopted by different researchers around the world [7]. The extensive range of natural polymers includes proteins (silk, collagen, gelatin, fibrinogen, etc.), polysaccharides (cellulose, amylose, dextran, chitin, and glycosaminoglycans), and polynucleotides (DNA and RNA) [2,8].

Chitosan [9,10], alginate [11,12,13], collagen [14,15], and different oligosaccharides are the most reliable options for scaffold’s synthesis based on natural polymers [6]. The importance of chitosan in the field of biomedicine is immense, thanks to its antimicrobial activity, biocompatibility, non-toxicity, biodegradability, and abundant availability around the world of its precursor (chitin), thus becoming very promising in tissue regeneration applications [16].

Different techniques used to prepare scaffolds are available to design sponges [17], membranes [18], hydrogels [9,19], meshes [20], and fibrous scaffolds [21]. The main objective of scaffold preparation is to get interconnectivity between the pores, facilitating the removal of waste from cells, and introduce nutrients from the environment [22]. Membranes based on nanofibrous biodegradable polymers and their structure-related cell adhesion and proliferation have been evaluated previously using in vitro assessments [23]. The use of chitosan-gelatin films for in vitro evaluation of fibroblast viability and proliferation demonstrated a determinant influence of gelatin blend on the cell behavior and scaffold properties [24].

The synthesis of the scaffolds based on chitosan and polyethylene glycol diacrylate hydrogel has been reported by the stereolithography technique with good long-term cell viability and spreading results [25]. Hydrogels based on carboxymethyl chitosan (CMC) and oxidized chondroitin sulfate (OCS) were prepared and loaded with bovine serum albumin (BSA) for in vitro degradation and BSA release of the CMs/gel scaffolds evaluation [26]. In vivo evaluation of chitosan-glycerol incorporating stem cells were evaluated for mandibular bone regeneration, exhibiting an exciting osteogenic potential [27]. Relevant literature for hydrogel and nanogels for biomedical applications has been published recently [28].

On the other hand, humanity in the ancient era learned the benefits of essential oils, which were frequently used. Thanks to their fungicidal, bactericidal, viricidal, insecticidal, and antiparasitic activity, they are widely applied in pharmacology even in our time. Additionally, in many cases, they have delightful aromas and flavors used in cosmetic applications due to their low toxicity, biocompatibility, and absence of allergic responses [29].

*Ruta graveolens* (rue) essential oil has a potent antimicrobial activity due to its coumarins, flavonoids, furanocoumarins, and alkaloids content. Besides, skin pharmaceuticals preparations from rue extracts have been studied with impressive results [30,31,32,33,34,35,36,37,38]. Despite that, several authors demonstrated that the 5- and 8-methoxypsoralen were active against *Rhizoctonia solanii*, *Fusarium* spp., *Pyrenochaeta lycopersici*, *Trichoderma viride*, *Penicillium* spp., *Thielaviopsis basicola*, and *Verticillium dahlia*, fungi which are also present in RGEO [39,40,41]. Antifungal activity of RGEO was also demonstrated using CS+RGEO coatings to control the fungal and quality decay of Guava [42].

Furthermore, although there is abundant literature on chitosan-based scaffolds, information related to scaffolds based on chitosan/essential oils is very scarce. A porous 3D scaffold of chitosan-gallic acid (CS/GA) essential oil was prepared via freezing and lyophilization, for tissue engineering applications [43]. CS/GA scaffolds (0.5–1.0%) showed 60–75% viability at 24 h and 90% at 48 h. SEM images showed that an increased cell attachment was observed for CS/GA scaffolds compared to CS scaffolds, demonstrating in vitro excellent cell viability and compatibility. Besides that, an important review was also published on the use of chitosan as the efficient delivery of essential oil systems for oral cavity care [44]. Another work reported blended membranes composed of chitosan and aloe vera gel through the solvent casting and was crosslinked with genipin to assessed in vitro cell viability with promising results [45].

Despite all the above, a deep emptiness remains for the application and biocompatibility evaluation in vivo conditions for CS/essential oil composites. For that reason, we evaluated the potential for short-term tissue engineering applications of CS+RGEO based on the biological activities of CS and RGEO.

## 2. Results and Discussion

### 2.1. Essential Oil Characterization

Chemical composition analysis of RGEO and the effect of preventing guava fungal and quality decay was reported previously [42]. The presence of biologically active compounds (Table S1) such as ketones compounds (76%), with 2-nonanone (23.5%) and 2-undecanone (42.6%), was evident. These results are consistent with those reported by Yaacob et al. [46], who demonstrated a high amount of 2-undecanone (30.73%), 2-nonanone (18.06%), and 2-nonyl acetate (11.03%) [46]. Undecan-2-one is the most common undecan-x-one within the plant kingdom. A significant amount of undecan-2-one is present in the essential oils from the *Rutaceae* family [47]. Several reports on biological activities of the RGEO are available, such as on larvicidal and nematicidal activities [33], antibacterial and antifungal potential [30,31,34,36], and allelopathy activity [35], indicating that chemical composition is crucial for biological activity. However, the synergistic effect between all the essential oil components could account for the biological activity observed in several essential oils [48]. Some components can interact with the cellular membrane creating holes, rendering the cell membrane more permeable and resulting in cell death, or sporulation and germination inhibition [49,50]. This antifungal activity observed was the primary motivation to include RGEO in chitosan-based film-forming solutions to obtain fast, porous, and resorbable films with antimicrobial benefits.

### 2.2. Physical-Chemical Characterization of the Film-Forming CS+RGEO Emulsions 

For a successful film formation, a stable film-forming emulsion should be prepared [51]. Table S2 shows the non-volatile fraction of the emulsion, which is constituted by chitosan and the essential oil components that could present strong interactions. The non-volatile concentration significantly increased (*p* < 0.05) with the RGEO content (2.86–3.87%) (F1–F3) except for (F4) RGEO 1.5% (3.59%), probably because the excess of oil-phase cannot be well-emulsified by the adsorbed chitosan chains and evaporates quickly under heat. Since the oil content increased, more chitosan chains will be adsorbed, and less amount will remain free in solution, significantly increasing the pH of the film-forming solution in an RGEO concentration-dependent manner [52]. In this work, the pH increased significantly (*p* < 0.05) from 4.36 to 4.43 (F1 to F4) (Table S2).

It is well known that among the most critical factors that determine the stability of an emulsion is the size of the drop, which also affects various properties of emulsions such as viscosity and rheological properties [51,53]. The protective electrostatic effect of the positively charged chitosan in acidic medium is due to its ability to adsorb at the water–oil interface, in addition to its high viscosity, preventing flocculation and cream formation by oil droplets [51]. 

On the other hand, the viscosity reduction of the emulsions with the RGEO incorporation could be a result of several factors. In this work, the viscosity decreased from 106 to 28.5 cp (F1 to F4) (Table S2). Chitosan amount in the continuous phase could suffer a decline as a result of the adsorption process on the oil–water interface, decreasing the thickening capacity [51,54]. However, with the increasing of the dispersed phase (RGEO), a reduction in the net electrical charge could occur as a result of the electrostatic interaction between the positively charged chitosan chains and negatively charged groups of different components of RGEO (ketones, phenols, and sesquiterpenoids), also decreasing the hydrodynamic volume of the CS particles. This electrical net charge reduction has been studied by ζ-potential and rheological tests, demonstrating a net charge and an electro-viscous reduction together with an increase in the particle size in an essential oil concentration-dependent manner [54]. As shown in Table S2, there was also an observed trend. Particle size increased with the RGEO incorporation from 1.0 to 1.6 µm (F1 to F4) (Table S2). However, the particle size was not affected significantly (*p* < 0.05) by the essential oil amount. The increasing of the particle size with the RGEO introduction could be a consequence of the lower amount of CS chains in the RGEO/water interfaces, which produce larger emulsion droplets with a reduction in the net electrical charge, as previously observed [55]. Similar results have been observed for emulsions incorporating basil, thyme, bergamot, lemon, *Thymus capitatus*, and tea essential oils [56,57]. 

### 2.3. Hemolysis Assay of the Essential Oil and Coatings of CS+RGEO.

Cell cytotoxicity can be understood as a modification in primary cellular functions due to a toxic agent. Different causes are possible for the hemolysis of erythrocytes. Among them, free radical destruction of the cells and microbial lysis is the most important, and essential oils could control both. Their components can act as antimicrobial molecules or radical scavengers, decreasing the erythrocyte hemolysis. One of the known cytotoxicity trials is the hemolysis test that involves the breakdown of red blood cells in the blood. Hemolytic assays were performed because compounds possessing cell adhesion and regeneration ability may not be useful in biomedical applications if they possess hemolytic effect [58]. 

The ability to carry water inside or outside the cell from the external medium is called tonicity. It determines the osmotic pressure to which red blood cells will be subjected, thus hypertonic or hypotonic media can destroy cells. Hypotonic media generate more exceptional transport of liquid into the cells, causing the blood to burst, while hypertonic media cause water loss in the blood. This process involves the release of the content present inside the plasma, which leads to the breakdown of the red blood cell. Therefore, the hemolysis percentage is useful to see the effect of an external agent on the blood erythrocytes. It has been previously demonstrated that several essential oils such as black pepper, cananga, and myrrh oils were able to decrease the hemolytic activity [59]. 

These results indicate that CS+RGEO emulsions are a useful free-radical scavenger and can modify the osmotic pressure. The protective effect (antioxidant or free-radical scavenger) of the essential oil depends on their hydroxyl groups amount, location, hydrophilic/hydrophobic balance (HLB), and the affinity with iron and hemoglobin groups [59].

**Figure 1:**
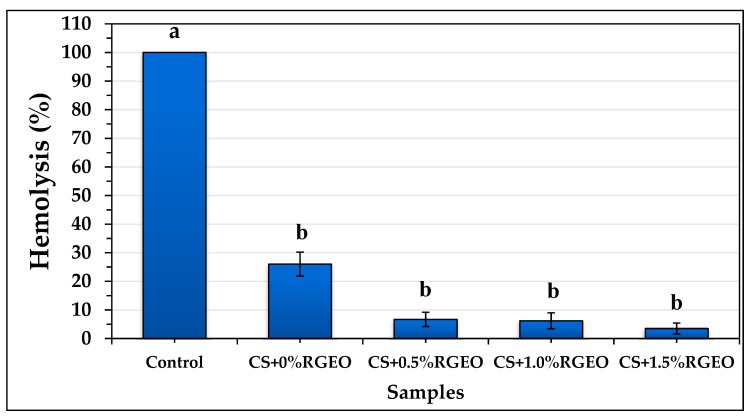
Hemolysis assay results (%) of the different formulations: CS (F1); CS+RGEO 0.5% (F2); CS+RGEO 1.0% (F3); and CS+RGEO 1.5% (F4). The means labeled with the letter “b” are significantly different from the mean of the control (*p* value < 0.05).

Normally, toxic molecules such as free-radicals and amphoteric molecules are produced in the human metabolic processes and cause erythrocytes lysis, which is controlled by the presence of natural antioxidants. Hemolysis in the presence or absence of CS+RGEO was performed and demonstrated a protective erythrocyte effect of the CS+RGEO emulsions. As shown in Figure 1, CS+RGEO inhibited hemolysis of human erythrocytes by 96.5% (CS+RGEO 1.5 wt.%), 93.8% (CS+RGEO 1.0 wt.%), 93.3% (CS+RGEO 0.5 wt.%), and 74% (CS without RGEO content). From the Figure S1 shows the protective effect of RGEO. RGEO inhibited hemolysis of human erythrocytes by 36%, while the water diluted samples of RGEO inhibited by 86.2%, 88.2%, and 91.9%, compared to the control. These results indicate that the RGEO could protect erythrocytes from hemolysis, while the combination with CS results in a protective synergistic effect.

A non-toxic effect of the essential oil is apparent, a necessary task for drug development and biomedical applications involved in the treatment of various diseases. Besides, the results reveal that the cytotoxic activity was not related to lytic properties or membrane instability induced by the emulsions [60]. It has been demonstrated that chitosan membranes containing tea essential oil can protect red blood cells. It is possible that positively charged amino groups present in the polymer matrix of the chitosan induce the agglomeration of the blood cells through the generation of electrostatic attractions with the negative charges present on the cell surface of erythrocytes, preventing damage of the cell membrane of red blood cells, reducing hemolysis [61]. All these results demonstrate a protective effect of the CS+RGEO emulsions of the human erythrocytes against hemolytic agents such as free radicals and ionic surfactants.

### 2.4. Physical-Chemical Properties of CS+RGEO Films

Biodegradable homogeneous films with yellowish translucid aspects were effectively produced by the casting method, as previously reported for similar chitosan-essential oil systems. Thicknesses were around 48–68 μm (Table 1), very similar to other reports [62]. However, the introduction of RGEO into the chitosan matrix produced several changes in the physical properties of CS films (Table 1). Thicknesses were increased significantly (*p* < 0.05) with the RGEO introduction in a concentration-dependent manner. The incorporation of the essential oil possibly produced some chain separation of the chitosan chains due to chemical interactions with RGEO components. According to Perdones et al. [63], greater solid contents per surface unit lead to increasing thicknesses.

On the other hand, moisture content did not decrease significantly (*p* < 0.05). A reduction in the availability of the chitosan amine and hydroxyl groups for water interactions by hydrogen bonding is the leading cause of moisture content affinity [64]. Despite that, the association for water only significantly decreased (*p* < 0.05) with the highest RGEO content incorporated, and lower solubility was observed (a reduction in water solubility, almost 43% from CS to CS+RGEO 1.5%) because of the interaction of RGEO components with CS chains. A similar fact was previously observed by García et al. [65]. 

Otherwise, CS+RGEO films were homogeneous, with a compact and smooth appearance. This compact structure could be the result of the interaction between CS chains and RGEO components, decreasing the water vapor coefficient (WVPC) (Table 1). As shown in Table 1, CS had a WVPC of 5.81 × 10^−10^ gs^−1^m^−1^Pa; however, after RGEO introduction, the WVPC significantly decreased (*p* < 0.05) as a result of the reduction in the hydrophilic character of CS chains and the physical interactions between CS and RGEO components that reduced water affinity. Water vapor permeability (WVP) will be affected if the porosity of the films is filled with essential oils, decreasing the spaces between the chains and water affinity (Table 1). The WVP reduction could result in a lower degradation or resorption of the materials in subcutaneous tissue. To solve these drawbacks, some researchers have introduced nanofillers such as nanoclays [66,67]. However, if the content is very resistant, low degradation and resorption will take place inside the body conditions, a severe drawback for short-term biomedical applications, such as wound-healing and dermal regeneration [68]. Nevertheless, the WVPC of CS films was higher than those obtained in other studies [69,70], while RGEO incorporation resulted in a decrease in the WVPC value.

### 2.5. Scanning Electron Microscope (SEM) Analysis of CS+RGEO Films

**Figure 2:**
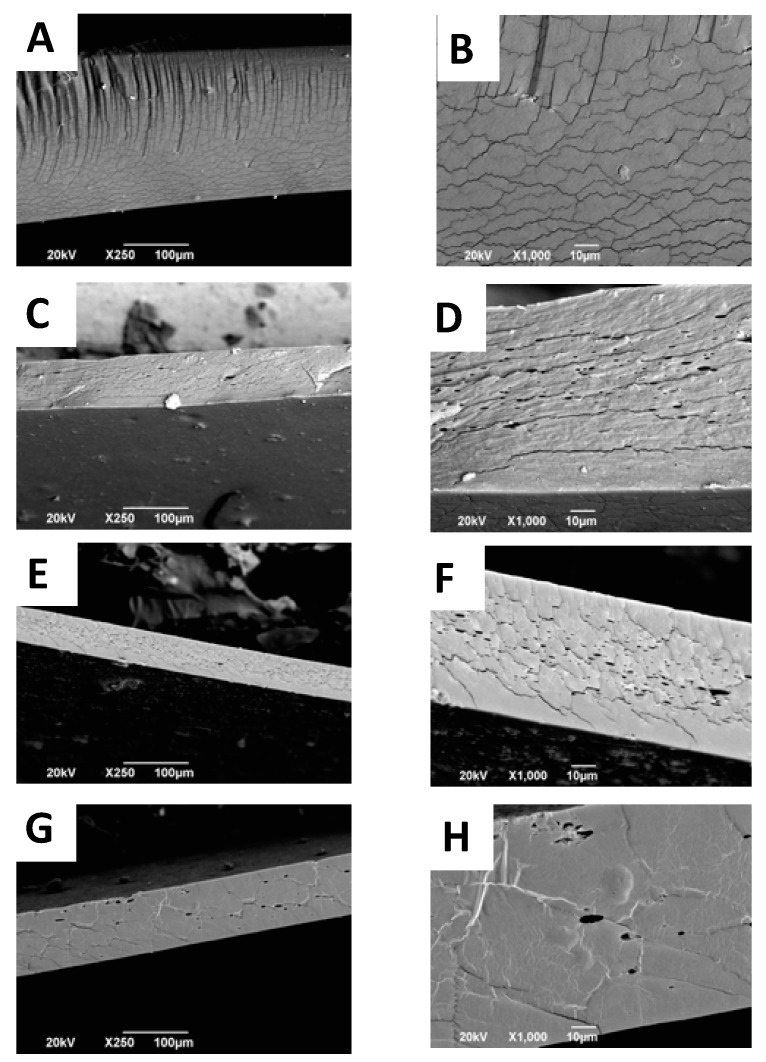
Cross-section analysis of the different formulations: CS (F1) (**A**,**B**), CS+RGEO 0.5% (F2) (**C**,**D**), CS+RGEO 1.0% (F3) (**E**,**F**), and CS+RGEO 1.5% (F4) (**G**,**H**), at ×1000 and ×250 of magnification, respectively.

The interaction between the essential oil components and the polymer matrix influences the microstructure of the films after the drying process. Furthermore, the structural arrangement of the different components of the film is influenced by various phenomena present such as coagulation, coalescence, creaming, and droplet coagulation, also determinant in mechanical behavior [54]. Surface analysis of CS films (Figure S3A,B) shows a flat and homogeneous surface, with some micro-fractures because of the essential oil presence (Figure S3C–H). When the oil content increases, more intense molecular interaction between chitosan and oil components occur, weakening the polymer chain aggregation forces, causing micro-fractures in the structure [53]. From the cross-sectional analysis (Figure 2), it is also possible to observe the porosity of the films by cause of the evaporation of RGEO and phase separation of the lipid components. However, with CS+RGEO 1.5%, a more compact structure is observed, which is also in agreement with the lower WVPC and lower water affinity. The denser structure could be a result of a homogeneous distribution of the RGEO and the drying process, possibly because a more economical creaming process occurs at higher lipid concentrations, as previously observed [54]. 

The size and the volume fraction of the lipid aggregates are the main factors creating heterogeneity in the film matrix [71]. Nevertheless, porosity is an advantage for tissue engineering applications, where a high-dense and interconnected structure is beneficial for cell adhesion and proliferation [72,73].

#### Color Analysis of CS+RGEO Films

Table 2 shows the analysis of L*, a*, and b* coordinates of the CS+RGEO films. The color analysis is very relevant for costumers since the color may cause rejection from consumers if it is not attractive [74]. The rectangular coordinates (L*, a*, and b*) and the total color difference (ΔE) of these films are recorded in Table 2. CS films are slightly yellow, which is indicated by the b* coordinate, normally observed for these CS-EO films [75]. However, the addition of the essential oil normally affects the coordinates [76]. In Table 2, it is observed that L* significantly (*p* < 0.05) decreased with the addition of RGEO, indicating lower lightness and brightness. These results could be a consequence of the chromophore content of RGEO, generating darker films. The addition of RGEO significantly increased the a* coordinate (*p* < 0.05) for 0.5% and 1.0% RGEO. However, the introduction of 1.5% of RGEO did not affect the parameter significantly, probably due to a saturation effect [75].

Finally, the b* coordinate increased significantly (*p* < 0.05) for all formulations except for CS+RGEO 1.5% (F4). The b* coordinate is related to the blue to yellow displacement color, which is also related to the introduction of the essential oil. Finally, there was a significant difference between the total difference in color (ΔE) of CS+RGEO 0.5% and CS+RGEO 1.0%. However, with higher amounts of RGEO (1.5%), there was no significant difference (*p* < 0.05), indicating that the RGEO introduction modifies the original color of the CS films until the saturation of the b* coordinate. The difference in colors is related to the chemical composition of the essential oils and the internal developed structure of the films after the drying process, which seems not to be affected after the introduction of 1.0% RGEO [71]. All the color changes observed in the present experiments are in accordance with previous observations of a decrease in film lightness and a shift in color towards red (+a*) and yellow (+b*) [76]. 

### 2.6. FTIR Analysis of CS+RGEO Films

**Figure 3:**
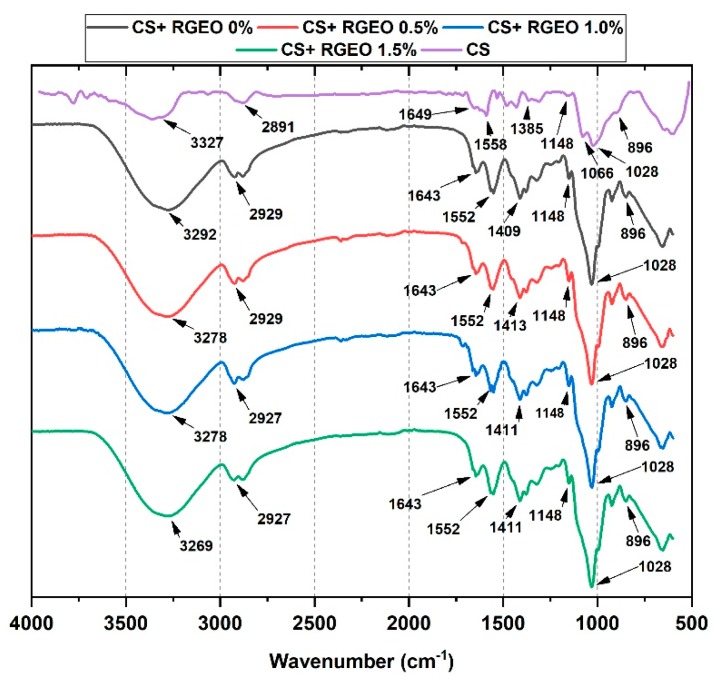
FTIR analysis of the different formulations: CS (F1); CS+RGEO 0.5% (F2); CS+RGEO 1.0% (F3); and CS+RGEO 1.5% (F4).

The FTIR characterization of the films is shown in Figure 3. The FTIR spectra of the different films show similar features between the formulations because of the CS content. The peaks at 896, 1028, 1066 (C=O stretching), and 1148 cm^−1^ (C-O-C bridge, glycosidic linkage) were related to CS and glycerol addition in all the films [77]. The small band associated with the amide-III was observed at 1385-1410 cm^−1^ (C-N stretching and N-H bending). The addition of glycerol to the chitosan solution increased the intensity of the band related to the C-O stretching vibration (1028 cm^−1^). Glycerol molecules likely displaced bound acetic acid from the chitosan structure. They thereby increased the number of the amino groups able to interact by hydrogen bonding with the glycerol molecules, shifting to lower values some amide bands such as those related to amide-I. In contrast, the O-H-related bands at 3000–3600 cm^−1^ increased the intensity due to the hydrogen bonding between chitosan chains and glycerol O-H groups [77].

On the other hand, the band at 1385 cm^−1^ was assigned to the acetamide groups of partially deacetylated CS. Bands at 1649 and 1558 cm^−1^ were attributed to C=O stretching (amide I) and N-H bending (amide II), respectively [78]. Interestingly, those bands increased their intensity with the RGEO incorporation as well as the increasing of the intensity of the C-H stretching vibration bands, a fact also observed by other researchers [78,79]. Besides, the bands at 1552 and 1643 cm^−1^ were higher, which might be due to the interaction of the amine and hydroxyl groups of chitosan with groups of the main components such as 2-nonanone and 2-undecanone of the essential oil. In addition, some shifting toward lower wavenumber values supports the interactions with the components of the essential oils, demonstrating excellent compatibility and interactions between CS and RGEO [78]. 

### 2.7. X-Ray Diffractometry (XRD) Analysis of CS+RGEO Films

**Figure 4:**
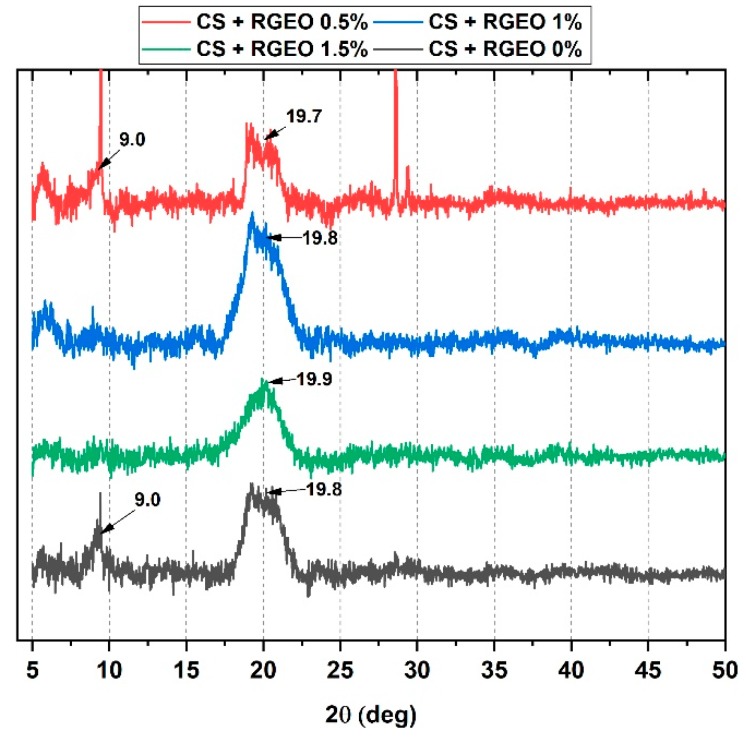
X-ray diffractometry (XRD) analysis of the different formulations: CS (F1); CS+RGEO 0.5% (F2); CS+RGEO 1.0% (F3); and CS+RGEO 1.5% (F4).

XRD analysis was performed to investigate the effect of RGEO introduction in the CS film crystallinity (Figure 4). CS film showed peaks at 2θ = 9.4, 19.8, and 20.9, which corresponded to the reflections 020, 200, and 220, respectively, of hydrated chitosan [77,80,81,82]. The CS film with glycerol and without RGEO exhibited the highest crystallinity as compared to the other films. The XRD pattern for CS and CS+RGEO 0.5% also showed a very weak diffraction peak located at 2θ = 9.0°, indicating higher crystallinity as compared to the other two formulations.

On the other hand, strong hydrogen bonding of CS with some components of the RGEO caused a shifting of the 2θ = 19.8° and the disappearing of 2θ = 9.4° for CS+RGEO 1.0% and CS+RGEO 1.5%, due to a possible loss in the crystallinity of the CS films. Similarly, Valenzuela, Abugoch, and Tapia [83] reported that the introduction of sunflower oil into the quinoa protein–chitosan matrix generated a less crystalline structure. At the same time, Hosseini et al. [84] stated an increase in the crystallinity of the chitosan nanoparticle films, including *Origanum vulgare* essential oil, an opposite effect that could be attributed to the nanoparticle distribution [83,84]. 

Usually, the crystallinity of the films depends on the components used to prepare the film-forming solution (including hydrocolloids, molecular weight, and deacetylation degree of the chitosan) and the dry process for the film-formation. In Table 3, it can be observed that the lowest crystallinity was obtained for F3 (CS+RGEO 1.0%). The new sharpening visible peaks at a 2θ = 9.0°, 2θ = 28.0, and 29.0° for F2 (CS+RGEO 0.5%) are possibly related to a better crystalline structure confirmed by the lower intensity of the 2θ = 19.7° peak, associated with the amorphous region of chitosan. This crystallinity improvement might be related to a better distribution of the RGEO in the polymer matrix due to a lower particle size generating a homogeneous and compact structure, also observed in the mechanical, microstructure, and thermal properties.

However, when more RGEO concentration was introduced to the films, there was a saturation effect that no longer improved crystallinity and mechanical properties, and, on the contrary, negatively affected them. 

To better understand the crystallinity of the films, we calculated, from the XRD and DSC curves, the crystallinity index (Table 3). Although the methodology by XRD is proposed for starch, it has also been adjusted for use in other types of polymers such as PVA [85] and CS compounds [86]. The percentage of crystallinity of the CS film (F1) is similar those reported by other researchers for CS/PVA films and confirms the semi-crystalline nature of this natural polymer. The results indicate that the addition of RGEO reduces the crystallinity of the material. This effect has been reported with the use of other essential oils such as *frankincense* oil [87] and *Carum copticum* essential oil [88]. It is attributed to the new interactions of CS and RGEO that slightly destroy the original crystalline structure of chitosan [77,88]. The crystallinity index by DSC is relative and is expressed concerning the CS (F1) film. Because of this, it has a value of 100%. The behavior of the crystallinity index by both techniques is quite similar.

### 2.8. Mechanical Properties of the Films

**Figure 5:**
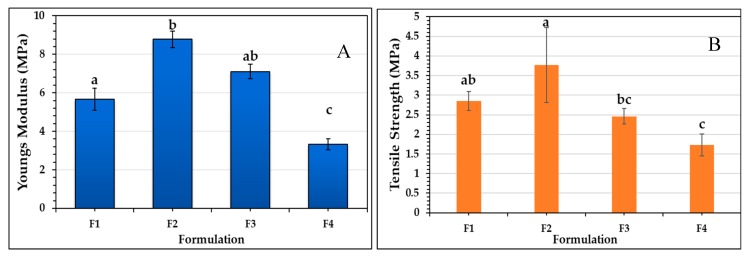
Mechanical properties study of the different formulations: CS (F1); CS+RGEO 0.5% (F2); CS+RGEO 1.0% (F3); and CS+RGEO 1.5% (F4). (**A**) Young’s modulus; and (**B**) tensile strength. Different lowercase letters mean statistical differences between the formulations (*p*-value < 0.05).

Biomedical application of biomaterials demands thermal and mechanical resistance with tissue compatibility [89]. Young’s modulus and the tensile strength were studied to evaluate the mechanical resistance of the different CS films. It can be observed in Figure 5 that RGEO introduction to the films significantly affected (*p* < 0.05) the tensile strength of the films. Initially, an increase from 2.9 ± 0.2 to 3.8 ± 1.0 MPa occurred between F1 and F2, but then, with the addition of 1.5% of RGEO (F4), the tensile strength significantly (*p* < 0.05) decreased to 1.7 ± 0.3 MPa. 

The same behavior can be evidenced in Young’s modulus, with the addition of the RGEO to the CS films. The incorporation of 0.5% of RGEO significantly increased (*p* < 0.05) the Young’s modulus from 5.7 ± 0.6 MPa to 8.8 ± 0.4 MPa, while the addition of 1.5% of RGEO significantly decreased (*p* < 0.05) the Young’s modulus to 3.3 ± 0.3 MPa. This result can be explained by an RGEO saturation phenomenon, in which an increase above 0.5% of RGEO causes an overload of essential oil. It promotes an increase in the mobility between the chains and, consequently, an increase in the flexibility of the films, decreasing the crystallinity and mechanical resistance. 

Other studies have also reported that complex structures appear between the lipid phase and the polymer, which can reduce the cohesion in the polymeric matrix, decreasing the tensile strength of the film [90]. 

This result is also consistent with the WVPC and XRD results. In WVPC, an increase in permeability is evident with the incorporation of 1.0% and 1.5% of RGEO, since intermolecular mobility facilitates the permeation of water vapor through the film. In addition, higher molecular movement is related to an amorphous and less crystalline structure, as evidenced in X-ray diffractometry for F3 and F4.

### 2.9. Thermal Analysis of CS+RGEO Films

#### Thermogravimetric Analysis (TGA) of the Films

**Figure 6:**
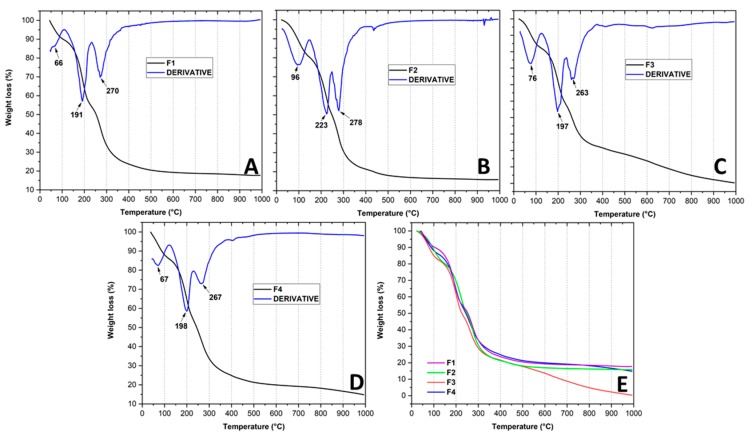
Thermogravimetric curves (TGA) of the different formulations: (**A**) CS (F1); (**B**) CS+RGEO 0.5% (F2); (**C**) CS+RGEO 1.0% (F3); (**D**) CS+RGEO 1.5% (F4); and (**E**) all the formulations together.

TGA curves presenting the thermal degradation of the CS and CS+RGEO films are shown in Figure 6. It is well known that CS films suffer degradation in three stages. However, with the introduction of RGEO, no changes in the degradation stages number occurred. 

Shen et al. reported up to five different degradation stages for chitosan films that included citronella essential oil and cedarwood oil [78]. The first stage (a mass loss of ~15%) represented a water and acetic acid residual loss as well as some volatile adsorbed compounds in the films that occurred at the approximate temperature range of 50–120 °C. 

The second degradation step (~30%) occurred within 190–250 °C and is attributed to the structural bound water as well as some degradation of chitosan, glycerol, and essential oil compounds. 

The final stage (~35%) occurred within 290–350 °C. A loss of saccharide units, dehydration, depolymerization, degradation of the more stable essential oil components, and loss of acetylated units could be the leading causes of the thermal behavior. As shown by the derivative curve in Figure 6 (blue line), the T_dmax_ for the three stages for the CS films incorporating RGEO had a slight increase, which means that a thermal reinforcement occurred, possibly due to hydrogen bonding with the essential oil components. 

The 1.0% RGEO formulation (F3) showed a fourth stage of weight loss above 350 °C (~20%) that could be related to the lower crystallinity observed for this formulation (Table 3). The lower crystallinity facilitates its degradation due to a higher disorder degree in the structure of the compound makes it easier for thermal decomposition.

Table 3 shows the melting temperatures for F1–F4. It is possible to see a low increase from F1 to F2 and from F2 to F3. However, for F3 and F4, no significant differences were observed, probably for the saturation effect observed for the mechanical, microstructure, and TGA analysis. However, the melting temperature increase for F2 agrees with the TGA analysis, where it is possible to see an increase in the degradation temperature for the second stage and with the mechanical properties study, and that Young’s modulus and Tensile strength showed the highest values for F2. With an introduction of 0.5% of RGEO, there was a reinforcement effect of the CS films, probably due to better distribution and compatibility with the polymer matrix. In addition, this result agrees with the smaller particle size and stability of the film-forming emulsion of the F2, which allows preparing a very stable and resistant film as compared to the other formulations. Finally, from Equation (9) analysis, it was also possible to study the crystallinity percentage of the films. The crystallinity decreased in an RGEO concentration-dependent manner, which agrees with XRD analysis. The introduction of the RGEO decreased the hydrogen bonding degree of CS chains because of the interaction with the oxygenated compounds of RGEO. Similar results have been observed in other studies [77]. 

### 2.10. In Vivo Studies 

Malafaya et al. [91] demonstrated by histological and immunohistochemistry findings that chitosan scaffolds can provide interconnectivity and to promote the neo-vascularization even in early stages of implantation [91]. We studied the histology of the films prepared after 30 days of implantation, with euthanasia of the Wistar rats performed and the recovery of the samples. Complete hair recovery was observed in the implanted area. In addition, skin shaving demonstrated a skin without continuity solutions and with healthy healing without signs of inflammation. On the other hand, a longitudinal incision with skin separation visualized the implanted sites, where small areas with remaining material were observed, without signs of inflammation or infection such as redness, presence of purulent exudate, or bad smell. 

**Figure 7:**
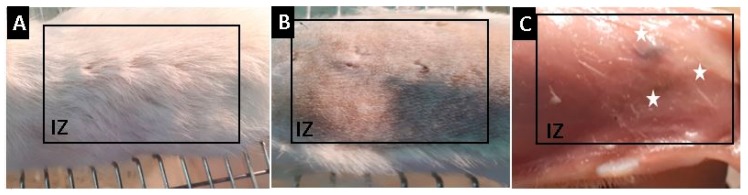
Macroscopic appearance of the Wistar’s rat skin implanted: (**A**) Formation of new skin; (**B**) Skin after shaving; and (**C**) Subdermal appearance of the skin. IZ, Implanted zone; Stars, Implanted material.

Figure 7 corresponds to the macroscopic appearance of the skin of the Wistar rats at 30 days of implantation.

**Figure 8:**
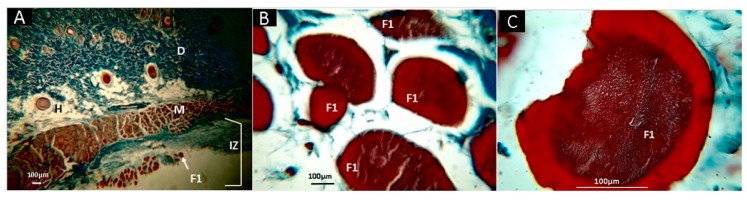
Histological analysis of sample F1 (CS). Magnifications are as follows: (**A**) 4× H-E; (**B**) 10× H-E; and (**C**) 40× H-E. D, dermis; H, hypodermis; IZ, Implantation zone; M, muscle; F1, formulation 1 (CS). Hematoxylin and eosin (H-E) technique.

Figure 8 corresponds to the histological analysis of the F1 films sub-dermally implanted after 30 days. In general, it is observed that the material shows some evidence of resorption on the surface (Figure 8B) and edges (Figure 8C). The capsule is made up of blue type I (CF) collagen fibers, evidenced by Masson’s Trichrome stain (MT). The inflammation process is typical for this type of sub-dermal process, without the presence of redness or pus, which demonstrates excellent compatibility with the tissue. Previously, it has been shown that chitosan with intact mineral content (17.9 wt.%), lowest molecular weight (11.49 KDa), and lowest deacetylation degree (83%) shows a well-structured subchondral bone and noticeable cartilaginous tissue regeneration implanted in rabbit knee osteochondral defects, and evaluated three months after surgery [92].

Early studies have demonstrated that chitosan induces enzymatic activity and phagocytosis [93,94]. Fujita et al. [95] synthesized hydrogels from chitosan able to degrade after 20 days of subcutaneous implantation [95]. Biocompatibility and low toxicity of chitosan are the main properties that support tissue compatibility and regeneration. 

Chitosan has potent cell adhesion ability depending on the deacetylation degree and composition. Scaffolds prepared from higher deacetylation degrees (>85) strongly supported the attachment and proliferation when compared with those made from lower deacetylated degrees with a composition of 2% (w/v) as in our study [92].

However, very few reports using composites of chitosan and essential oils have been presented, especially for in vivo studies. In vitro results have been more common to evaluate the biocompatibility of chitosan. For example, CS/GA scaffolds (0.5–1.0%) showed 60–75% viability at 24 h and 90% at 48 h. In addition, the images show an increased cell attachment for CS/GA scaffolds compared to CS scaffolds [43], demonstrating excellent cell viability and in vitro compatibility when chitosan-essential oil composites are used for tissue engineering applications.

**Figure 9:**
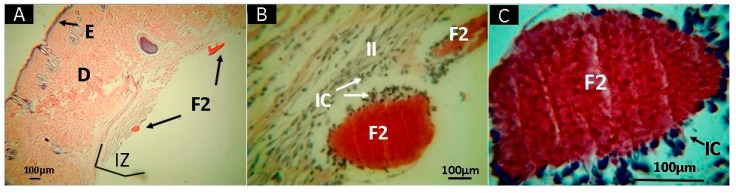
Histological analysis of sample F2 (CS+RGEO 0.5%). Magnifications are as follows: (**A**) 4× H-E; (**B**) 10× H-E; (**C**) 40× H-E. D, dermis; H, hypodermis; M, muscle; IZ, Implantation zone; II, Inflammatory infiltrate; IC, Inflammatory cells; F2, Formulation 2 (CS+RGEO 0.5%). Hematoxylin and eosin (H-E) technique.

On the other hand, the material corresponding to F2 (CS+RGEO 0.5%), presented almost complete resorption of the samples after 30 days of subdermal implantation, with the persistence of some small fragments in the implantation zone (IZ), as shown in Figure 9A. At 10× magnification, it is possible to see that these fragments are surrounded by an inflammatory infiltrate (II) (Figure 9B) with several inflammatory cells (IC) phagocyting the material, which is better observed at a higher magnification (40×, Figure 9C). 

These findings are exciting because they demonstrate that RGEO facilitates the resorption process. It is not clear the reason, but it could be due to a stimulation of the phagocytosis mechanism and the higher porosity of the material after RGEO evaporation. More studies are necessary to understand the mechanism of the resorption and degradation occurring. It has been demonstrated that a higher number of cells that adhered to the surface in CS-GA mixtures could be a result of the blending of GA with CS, which shields the highly positive charge density of CS, hence increasing cell attachment [43].

**Figure 10:**
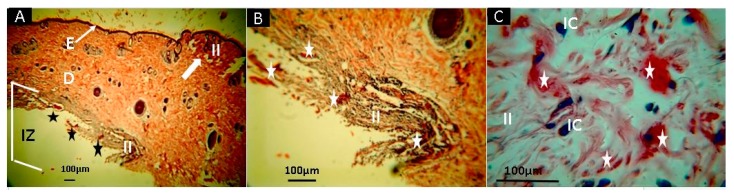
Histological analysis of sample F3 (CS+RGEO 1.0%). Magnifications are as follows: (**A**) 4× H-E; (**B**) 10× H-E; and (**C**) 40× H-E. D, dermis; E, epidermis; white arrows, continuity zone to the epidermis; H, hypodermis; M, muscle; IZ, Implantation zone; II, Inflammatory infiltrate; IC, Inflammatory cells; Stars, the implanted material; F3, formulation 3 (CS+RGEO 1.0%). Hematoxylin and eosin (H-E) technique.

Samples of F3 are observed in Figure 10. F3 (CS+RGE0 1.0%) had more significant resorption compared to F1 and F2. On the other hand, there is remaining material in the middle of an inflammatory infiltrate (II). These results demonstrate a higher degradation/resorption amount of content, producing more inflammatory processes. The inflammatory process is typical in the healing process, and the absence of pus demonstrates the efficiency of the scaffolds to support cell adhesion. These formulations have the synergistic combination of a strong antimicrobial effect of RGEO components with the antimicrobial and biocompatibility properties of CS, as previously pointed [96].

**Figure 11:**
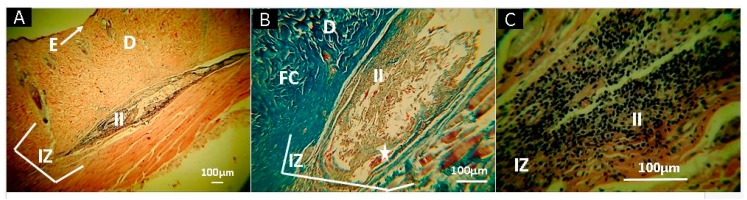
Histological analysis of sample F4 (CS+RGEO 1.5%). Magnifications are as follows: (**A**) 4× H-E; (**B**) 10× MT; and (**C**) 40× H-E. D, dermis; E, epidermis; w hite arrows, continuity zone to the epidermis; IZ, Implantation zone; II, Inflammatory infiltrate; FC, Fibrous capsule; Stars, the implanted material; F4, formulation 4 (CS+RGEO 1.5%). Hematoxylin and eosin (H-E) and Masson’s trichrome stain (MT) techniques.

F4 (Figure 11), unlike the other formulations, had a highly localized implantation area with almost no remaining content (almost completely resorbed), but with abundant inflammatory infiltrate (II). The previous result agrees with a natural degradation by the higher water permeability and lower mechanical properties presented for this formulation.

This finding supports that higher amounts of RGEO (>0.5%) will produce higher porosity of the films, but also a higher immune response from IC as a response to the presence of RGEO components. However, from the cytotoxic tests, we can conclude that no erythrocytes cytotoxic reactions are present. These results are impressive, demonstrating the application of the CS+RGEO for cell adhesion and proliferation, preferable at low RGEO concentrations (<1.0%). However, different cell lines need to be tested to assure that RGEO components are not toxic to humans at higher levels.

## 3. Materials and Methods 

### 3.1. Composition of Essential Oil of Ruta graveolens

The essential oil of *Ruta graveolens* (Krauters, Bogotá, Colombia) and its composition were analyzed by gas chromatography–mass spectrometry (GC-MS) (Agilent Technologies, Inc, Santa Clara, USA) using an AT 6890 Series plus gas chromatography spectrometer, with a mass selective detector (full scan), as reported previously [42]. 

### 3.2. Emulsion Preparation and Characterization 

Chitosan (from shrimp shells) with a molecular weight of Mv 144.000, measured by capillary viscometry (using an Ubbelohde 0C viscometer), was used for the preparation of the film-forming emulsions. The reported values of K and a for CS in the solvent used (acetic acid 0.3 M + sodium acetate 0.2 M) at 25 °C are 0.074 mL/g and 0.76, respectively [97], for the calculation of the molecular weight using Equation (1) (the Mark–Houwink–Sakurada Equation):

(1) $$\left[\eta \right]=K{\left(Mv\right)}^{a}$$

The deacetylation degree of the CS (89−90%) was determined by ^1^H-NMR (using a BRUKER AVANCE II spectrometer with 400 MHz of frequency at a temperature of 300K) and elemental analysis. The sample was dissolved in D_2_O with two drops of trifluoroacetic acid as solvent and 3- (trimethylsilyl)propionic acid-d_4_, as reference salt. Finally, the elemental analysis was performed using a Thermo Electron Flash EA 1112 equipment). Tween 80, glycerol, and glacial acetic acid were purchased from Sigma-Aldrich (Palo Alto, California, United States) and used as received to prepare the emulsions. Briefly, RGEO was introduced to a 2% (*w*/*v*) CS (acetic acid 1% *v/v*) solution in different concentrations (0.5%, 1.0%, and 1.5% *v*/*v*) [42]. The different formulations are: CS (F1); CS+RGEO 0.5% (F2); CS+RGEO 1.0% (F3); and CS+RGEO 1.5% (F4).

#### 3.2.1. Droplet Size 

The droplet size of the CS+RGEO emulsions was performed with a previously reported method [57], using an AIMSIZER 2011 laser diffractometer (Dandong Liaoning, China) under International Organization for Standardization (ISO standard) [98].

#### 3.2.2. Viscosity Measurements

A Brookfield LVF viscometer (Toronto, Canada) was used at a temperature of 25.0 ± 0.2 °C in a beaker, according to the ASTM D2196-99 standard [99]. 

#### 3.2.3. Total Solid Content 

It was determined as the reported methodology [57], according to Equation (2):

(2) $$\%S=\left(\frac{P_{s}-P_{d}}{P_{m}-P_{d}}\right)\times 100$$

where *%S* is the (*w*/*w*) percentage of solids, *P_d_* is the weight of dry and clean aluminum disk (g), *P_m_* is the weight of the aluminum disk containing sample (g), and *P_s_* is the weight of the dry sample plus the aluminum disc (g).

### 3.3. Hemolysis Assay

For the hemolysis determination, a reported methodology was followed with some modifications. For the collection of the blood, samples with anticoagulant EDTA (ethylenediaminetetraacetic acid) were used. The blood donor was a 20- year-old male with AB+ group blood. The sample was centrifuged at 350× *g* for 5 min. The blood plasma was discarded to obtain the erythrocytes. The red cells were subjected to three washes with 3 mL of 0.9% (w/v) saline solution and centrifuged at 350 g for 5 min. With an isotonic solution of PBS (phosphate-buffered saline) at a pH of 7.4, a suspension of erythrocytes-PBS at 5% (*v/v*) was prepared. To determine the percentage of hemolysis, 28 μL of red cell solution, 3.958 μL of PBS, and 14 μL of each sample were added to the emulsions (T1 = CS, T2 = CS + RGEO 0.5%, T3 = CS + RGEO 1.0%, and T4 = CS + RGEO 1.5%). The control sample for total hemolysis consisted of 3.972 μL of sterile deionized water with 28 μL of erythrocyte solution.

Samples of essential oil without chitosan (see Supplementary Materials) were prepared using 0.2% (*v/v*) of Tween 80 and the RGEO was diluted in sterile deionized water to obtain the desire concentration (100% RGEO, 1.5% RGEO, 1.0% RGEO, and 0.5% RGEO). Each sample was placed in Falcon tubes, which were placed in continuous shaking at 90 rpm in a Shaker and 37 ± 0.2 °C. Centrifugation at 700 g of the samples was performed for 1 h, and the supernatant absorbance was determined at 540 nm. The test was done by triplicate. The percentage of hemolysis was calculated using Equation (3):

(3) $$\%\;Hemolysis=\left(\frac{SA}{THA}\right)\times 100$$

where *SA* is sample absorbance and *TAH* is the total absorbance of hemolysis.

### 3.4. Physicochemical Characterization of CS+RGEO Films

#### 3.4.1. Film Characterization

Thickness analysis of CS+RGEO films was determined using a digital Mitutoyo Digimatic micrometer (coolant proof micrometer ip65) to the nearest 0.001 mm.

#### 3.4.2. Film Solubility in Water 

Pieces of a film of 2 cm^2^ were cut and dried at 105 °C to constant weight. The solubility was measured from immersion assays under continual agitation in 50 mL of deionized water for 24 h at 25 °C. The initial dry weight and remaining pieces of the film after 24 h of immersion were dried at 105 °C to constant weight (Equation (4)).

(4) $$\%\;Solubility\;in\;water=\left(\frac{initial\;dry\;weight-final\;dry\;weight}{initial\;dry\;weight}\right)\times 100$$

#### 3.4.3. The Moisture Content of CS+RGEO Films

Pieces of a film of 2 cm^2^ were cut from each film, at 105 °C to constant weight to obtain the final dry weight. The moisture content was calculated according to Equation (5):

(5) $$\%\;moisture\;content=\left(\frac{initial\;wet\;weight-final\;dry\;weight}{initial\;wet\;weight}\right)\times 100$$

#### 3.4.4. Water Vapor Permeability

The water vapor transmission was determined gravimetrically by following the method E96-05 (ASTM, 2005) [100]. A glass permeation cell filled with silica gel (0% RH) was employed for test. The film sample with a diameter of 80 mm was sealed with silicone on a circular cell mouth. The cell was stored in an airtight container containing a saturated saline solution of sodium chloride (73 ± 2% RH) at 25 °C. Changes in weight of the permeation cell were plotted as a function of time, and each line slope was calculated by linear regression. Wvt (g/Pa s m) was calculated according to Equation (6):

(6) $$Wvt=\left(\frac{WVTR}{P}x\;RH\right)\times 100$$

where *WVTR* is the water vapor transmission rate, calculated as the ratio between the slope of the straight line (g/s) and the permeation of the cell area (m^2^); *p* is the saturation vapor pressure of water (Pa); *RH* is the relative humidity in the airtight container; and l is the average film thickness (m). Analyses were conducted in triplicate.

#### 3.4.5. Color Analysis of CS+RGEO Films

Film color was determined using a Konica Minolta CM 600d^®^ colorimeter (CR-300, Tokyo, Japan). The white tile was used as standard during the color measurement. Lightness (L*) and chromaticity parameters a* (red to green) and b* (yellow to blue) were used to characterize the film color in the Hunter Lab-scale (CIE Lab scale). The total color difference (ΔE) was calculated using Equation (7).

ΔE = ([ΔL*]^2^ + [Δa*]^2^ + [Δb*]^2^)^0.5^ (7)

where ΔL = L*standard–L*sample, Δa = a*standard—a*sample, and Δb = b*standard—b*sample. The standard refers to the control sample (chitosan film with 0% of RGEO).

#### 3.4.6. Fourier-Transform Infrared Spectroscopy (FTIR)

FTIR studied the chemical identity of functional groups in ATR mode (attenuated total reflectance) with a diamond tip (Shimadzu, Kyoto, Japan).

#### 3.4.7. X-Ray Diffraction (XRD)

The characteristic bands of the X-ray spectrum of CS-RGEO films were taken on a PANalytical X’Pert PRO diffractometer (Malvern PANalytical, Jarman Way, Royston, UK) using Cu Kα1 radiation (1.540598 Å) and Kα2 (1.544426 Å), with an electron accelerator voltage of 45 kV, an electron-generating current of 40 mA, an optical grid of incident beam 1°, and a diffracted beam grid of 9.1 mm, in a range 2θ between 5° and 40°. 

According to Nara–Komiya methodology [101], the crystallinity percentage from XRD technique (Xc XRD %) was calculated using Equation (8):

(8) $$X_{c}\left(\%\right)=\left(\frac{A_{c}}{A_{T}}\right)\times 100$$

where *A_C_* is the area under the peaks that represents the crystalline region and *A_T_* is the total area of the crystalline and amorphous region.

#### 3.4.8. Scanning Electron Microscopy (SEM)

The surface and cross-sectional microstructure were analyzed using a scanning electron microscope (SEM) (JEOL JSM-6490LA, Musashino, Tokyo, Japan). The voltage applied was 20 kV with the mode of secondary backscattered electrons. For the proper conductivity of the samples, a coating of gold was prepared.

#### 3.4.9. Mechanical Studies

Mechanical studies were performed in a universal SHIMADZU EZ-LZ test machine (Shimadzu, Kyoto, Japan) under the ASTM D882 standard. Six specimens per formulation were used. The gap between jaws was 100 mm, the width of the film was 20 mm, and the test speed was 50 mm/min.

#### 3.4.10. Thermal Studies

Thermal gravimetric analysis (TGA) was performed on a TA Instruments TGA Q50 V20.13 Build 39 (TA instrument, Delaware, New Castle, USA). The samples were heated up to 1000 °C at a heating rate of 10 °C/min under nitrogen atmosphere (flow rate 80 mL/min). DSC cycle heating was performed between 25 and 350 °C and again cooling to 25 °C. The fusion temperature (Tm) was determined by differential scanning calorimetry using a DSC2A-00181 (TA instrument, Delaware, New Castle, USA) from the heating at 10 °C/min. TGA and DSC data were analyzed using TA Instruments’ Universal Analysis software.

According to Noor et al. [85], the crystallinity percentage from the DSC technique (Xc DSC %) was calculated using Equation (9):

(9) $$X_{C\;}\left(\%\right)=\left(\frac{\Delta H_{m}}{\Delta H_{0}}\right)\times 100$$

where Δ*H_m_* is the fusion enthalpy of the CS compound and the essential oil and Δ*H_o_* is the enthalpy of fusion of the CS compound.

### 3.5. In Vivo Studies

The immune response of the composite films (F1–F4) was studied using Wistar rat subcutaneous tissue implantation, as previously reported [68,102,103,104]. Composite films of 5 mm of diameter were implanted in subdermal position according to ISO 10993-6. Commercial porcine collagen was used as a control, and the sedative was a solution of ketamine (70 mg/kg) and Xilacin (30 mg/kg) from Laboratory HOLLIDAY SCOTT S (Buenos Aires, Argentina) under the intramuscular application. Eight-month-old Wistar rats were supplied by the Bioterio of the Universidad del Valle. This research was reviewed, supported, and supervised by the Institutional Ethics Review Committee with experimental animals of the Universidad del Valle (CEAS 012-019).

After 30 days of implantation, the samples were recovered, fixed in buffered formalin, and dehydrated in alcohol solutions of ascending concentration (70%, 80%, 95%, and 100%). In addition, samples were diaphanized with xylol and infiltrated with paraffin for later cutting to 5 µm using a Thermo Scientific^TM^ Histoplast Paraffin™ and an Autotechnicon Tissue Processor™ (Leica Microsystems, Mannheim, Germany). For the image analysis, a Leica Microscope with a camera Leica DFC and the application Leica Suite 4.12.0 (Leica Microsystems, Mannheim, Germany). The samples studied for histological analysis used hematoxylin and eosin (H-E) and Masson’s trichrome stain (MT) techniques.

### 3.6. Statistical Analysis

Minitab statistical software (Version 17) was employed to determine the analysis of variance (ANOVA) and the comparison of the average, with a confidence level of 95% (α = 0.05). Dunnett test was used to evaluate the effect of hemolysis of CS+RGEO emulsions. Fisher test was used to assess the impact of color analysis and physical and mechanical properties. 

## 4. Conclusions

The successful incorporation of *Ruta graveolens* essential oil into the chitosan matrix was demonstrated using FTIR, and changes in the crystallinity, thermal behavior, and physical-chemical properties were observed. CS+RGEO films exhibited lower water permeability, water solubility, and comparable thermal resistance than CS films, except for 0.5% of RGEO, where a thermal reinforcement effect was shown by TGA and DSC (fusion temperature), but with a saturation point when an excess of 1.5% of RGEO was used. The analysis of Young’s modulus and tensile strength showed a decrease in the values according to the RGEO incorporation, except for 0.5%, where there was an increase in the values due to smaller particle size and better distribution. However, increasing the RGEO amount resulted in a loss of the crystallinity percentage of the CS films. The microstructure analysis of the cross-section of the films demonstrated that the homogeneous and compact structure changed with the RGEO introduction, generating porosity and cracking in the films. These results are an advantage for tissue engineering applications where cell adhesion and proliferation require porosity and resorbable structures. Finally, in vivo implantation studies in Wistar rats demonstrated very good resorption of the films, especially for those with higher RGEO content, but with abundant inflammatory infiltrate. This finding supports that higher RGEO percentages (>0.5%) will produce higher porosity and cracking of the films but also a higher immune response that could be affected by the presence of RGEO components and lower thermal and mechanical properties. However, from the cytotoxic tests, we can conclude that no allergic or cytotoxic reactions in erythrocytes were present. These results are impressive, demonstrating the application of the CS+RGEO for cell adhesion and proliferation, preferable at low RGEO concentrations (<1.0%).

## Figures and Tables

**Table 1:** Physical properties of the CS+RGEO films.

Formulation	Thickness (µm)	Moisture Content (%)	Solubility in Water (%)	Water vapor Permeability Coefficient (gs^−1^m^−1^Pa × 10^−10^)
F1	48.32 ± 0.02 ^a^	42 ± 5 ^a^	28 ± 5 ^a^	5.81 ± 0.07 ^a^
F2	52.11 ± 0.05 ^b^	42 ± 2 ^a^	26 ± 1 ^a^^,b^	1.60 ± 0.02 ^c^
F3	59.34 ± 0.01 ^c^	43 ± 1 ^a^	24 ± 11^a^	2.29 ± 0.04 ^b^
F4	66.75 ± 0.02 ^d^	41 ± 1 ^a^	15 ± 7 ^b^	2.04 ± 0.07 ^b,c^

* Values correspond to means ± standard deviation. Different superscript letters in the same column indicate significant differences (*p* < 0.05). The different formulations: CS (F1); CS+RGEO 0.5% (F2); CS+RGEO 1.0% (F3); and CS+RGEO 1.5% (F4).

**Table 2:** Color analysis of CS+RGEO films.

Sample	L*	a*	b*	a*/b*	ΔE
F1	58 ± 1 ^a^	−1.2 ± 0.1 ^a^	9 ± 2 ^a^	−0.14 ^a^	-
F2	54 ± 3 ^b^	−0.9 ± 0.3 ^a,b^	12 ± 4 ^b^	−0.085 ^b^	6 ± 4 ^a^
F3	50 ± 2 ^c^	−0.4 ± 0.6 ^c^	16 ± 3 ^c^	−0.030 ^c^	10 ± 3 ^b^
F4	52 ± 2 ^b,c^	−0.6 ± 0.7 ^b,c^	18 ± 5 ^c^	−0.043 ^b,c^	11± 5 ^b^

Note: Lightness (L*) and chromaticity parameters a* (red to green) and b* (yellow to blue) were used to characterize the film color in the Hunter Lab-scale (CIE Lab scale). ΔE is the total color difference. Values are expressed as mean ± standard deviation. Different letters in the same column indicate significant differences (*p* < 0.05). The different formulations: CS (F1); CS+RGEO 0.5% (F2); CS+RGEO 1.0% (F3); and CS+RGEO 1.5% (F4).

**Table 3:** The crystallinity index (Xc) and melting temperature (Tm) of the different formulations calculated from XRD and DSC analysis.

Formulation	Xc XRD (%)	Xc DSC (%)	Tm (°C)
F1	10.9	100	247.3
F2	10.7	78.6	254.4
F3	5.0	78.2	250.1
F4	6.4	87.6	252.9

## Supplementary Materials

The following are available online, Table S1. Volatile compounds identified in *Ruta graveolens* essential oil. Table S2. Physical-chemical properties of the CS+RGEO coatings. Figure S1. Hemolysis assay results (%) of the different formulations: CS (F1); CS+RGEO 0.5% (F2); CS+RGEO 1.0% (F3); and CS+RGEO 1.5% (F4). Figure S2. Surface electron microscopy analysis of the different formulations: CS (F1); CS+RGEO 0.5% (F2); CS+RGEO 1.0% (F3); and CS+RGEO 1.5% (F4). F1 (A,B); F2 (C,D); F3 (E,F); F4 (G,H); (A,C,E,G) at ×250 of magnification; and (B,D,F,H) at ×1000 of magnification. Figure S3. Crystallinity analysis from XRD curves of the different formulations: CS (F1); CS+RGEO 0.5% (F2); CS+RGEO 1.0% (F3); and CS+RGEO 1.5% (F4). F1 (A,B); and F2 (C,D).
