# Supplementary material for: Synthesis, Characterization, and Histological Evaluation of Chitosan-Ruta Graveolens Essential Oil Films
Source: Molecules. 2020 Apr 7;25(7):1688. doi: 10.3390/molecules25071688 (PMC7180789; doi:10.3390/molecules25071688)
Supplement: Supplementary file 1 [file molecules-25-01688-s001.pdf]

Supporting information

Article

Synthesis, Characterization, and Histological Evaluation of Chitosan-Ruta Graveolens Essential Oil Films

Carlos David Grande Tovar [1], Jorge Iván Castro [2], Carlos Humberto Valencia Llano [3], Diana Paola Navia Porras [4], Johannes Delgado Ospina [4], Mayra Eliana Valencia Zapata [5], José Herminsul Mina Hernandez [5,*] and Manuel N. Chaur [2,6,*]

1 Grupo de investigación de fotoquímica y fotobiología, Universidad del Atlántico, Carrera 30 Número 8-49, Puerto Colombia 081008, Colombia; carlosgrande@mail.uniatlantico.edu.co
2 Grupo de Investigación SIMERQO, Departamento de Química, Universidad del Valle, Calle 13 No. 100-00, Cali 76001, Colombia; jorge.castro@correounivalle.edu.co
3 Escuela de Odontología, Grupo biomateriales dentales, Universidad del Valle, Calle 4B # 36-00, Cali 76001, Colombia; carlos.humberto.valencia@correounivalle.edu.co
4 Grupo de Investigación Biotecnología, Facultad de Ingeniería, Universidad de San Buenaventura Cali, Carrera 122 # 6-65, Cali 76001, Colombia; dpnavia@usbcali.edu.co (D.P.N.P.); jdelgado1@usbcali.edu.co (J.D.O.)
5 Escuela de Ingeniería de Materiales, Facultad de Ingeniería, Universidad del Valle, Calle 13 No. 100-00, Santiago de Cali 760032, Colombia; valencia.mayra@correounivalle.edu.co
6 Centro de Excelencia en Nuevos Materiales (CENM), Universidad del Valle, Calle 13 No. 100-00, Santiago de Cali 760032, Colombia
* Correspondence: jose.mina@correounivalle.edu.co (J.H.M.H.); manuel.chaur@correounivalle.edu.co (M.N.C.); Tel.: +572-3212100

Table S1. Volatile compounds identified in *Ruta graveolens* essential oil [1].

Chemical function	Compound	RT	Amount Relative (%)	KI
Alcohol	2-undecanol	31.45	1.1	1304
	Manol	52.46	0.5	2076
	2-nonanol	23.84	3.0	1102
	1-nonanol	26.55	0.1	1172
Ketone	α-Thujone	24.25	0.1	1113
	2-undecanone	31.15	42.6	1296
	2-octanone	19.1	0.2	990
	2-decanone	27.38	4.0	1193
	(*R*)-(-)-Carvone	29.52	0.1	1251
	2-Dodecanone	34.93	2.9	1396
	2-nonanone	23.48	23.5	1094
	2-Tridecanone	38.44	2.5	1497
Ester	Octyl acetate	27.99	0.2	1209
	Benzyl acetate	46.24	1.7	1782
	1-Methylheptyl acetate	28.82	1.3	1232
	trans-farnesyl acetate	47.73	0.2	1834
	Benzyl 2-hydroxybenzoate	48.61	0.5	1887
Sesquiterpene	Nonyl acetate	31.62	0.7	1309
	Isodecanone	33.78	2.6	1366

	Geijerene	25.65	0.1	1149
	Isogeijerene C	29.98	0.1	1264
	Cogeijerene	30.36	0.2	1274
	Tetradecane	35.17	<0.1	1402
	Cis-β-Caryophyllene	35.7	0.1	1417
	Methyldecyl acetate	36.09	0.2	1429
	trans-β-Caryophyllene	36.28	0.8	1434
	(-)-Aromadendrene	36.53	0.9	1442
Sesquiterpene	Allo-aromadendrene	36.72	0.2	1447
	Isotridecanone	37.2	0.4	1461
	α -Humulene	37.53	1.1	1470
	γ -Muurolene	38.05	0.3	1485
	Geijerene	25.65	0.1	1149
	Valencene	38.64	0.2	1503
	α-Farnescene	38.75	0.2	1506
	γ -cadinene	39.31	0.2	1525
	σ-cadinene	39.41	0.5	1528
	α-Farnescene	43.44	0.2	1670
	(+)-cubenene	39.9	0.1	1545
Sesquiterpenoid	Viridiflorol	41.87	0.8	1611
	β-Eudesmol	43.52	0.2	1673
	Trans-Farnesol	44.72	0.3	1719
Furocoumarin	Ficusin	47.76	0.2	1849
	Chalepensin	54.8	1.1	2196
	N.I. (M+ 162)	29.76	0.9	1258
	N.I. (M+ 160)	43.61	0.3	1676
	N.I. (M+ 186)	43.7	1.1	1680
	N.I. (M+ 232)	47.25	1.0	1826
	N.I. (M+ 248)	51.94	0.4	2049
	N.I. (M+ 180)	52	0.1	2052

* KI is the Kováts Retention Index relative to C5–C24 n-alkanes on the. DB-5 column.

Table S2. Physical-chemical properties of the CS+RGEO coatings [1].

**Essential Oil (%)**	**pH***	**Density* (g/mL)**	**Apparent viscosity* (cP)**	**Solids* (%)**	**Particle Size* (µm)**
0	4.38 ± 0.01^{a}	1.002 ± 0.006^{a}	106.0 ± 0.1^{d}	2.56 ± 0.02^{a}	N.D.
0.5	4.40 ± 0.01^{b}	1.008 ± 0.005^{a}	74.0 ± 0.1^{c}	3.71 ± 0.01^{b}	1.0 ± 0.3^{a}
1.0	4.41 ± 0.01^{c}	1.008 ± 0.008^{a}	66.0 ± 0.1^{b}	3.87 ± 0.02^{c}	1.2 ± 0.3^{a}
1.5	4.43 ± 0.01^{d}	1.009 ± 0.006^{a}	28.5 ± 0.2^{a}	3.59 ± 0.02^{d}	1.6 ± 0.1^{a}

* Values correspond to means ± standard deviation. Different superscript letters (a,b,c,d) in the same column indicate significant differences between treatments ($p < 0.05$). N.D.= Not determined.

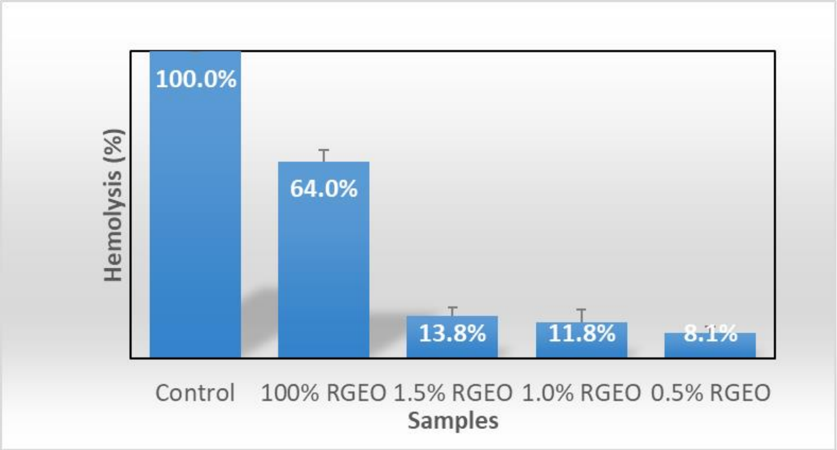

Figure S1. Hemolysis assay results (%) of RGEO diluted in water.

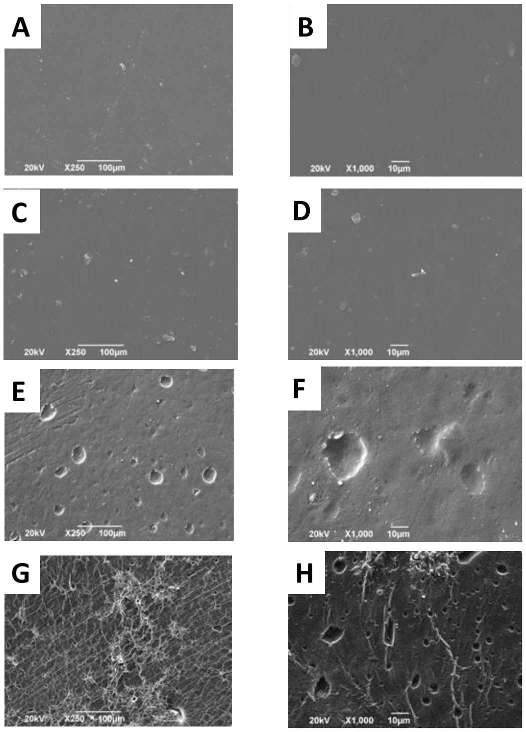

Figure S2. Surface electron microscopy analysis of CS+RGEO films with 0% (**A**) 0.5% (**B**), 1.0% (**C**) and 1.5% (**D**), at ×1000 of magnification, respectively.

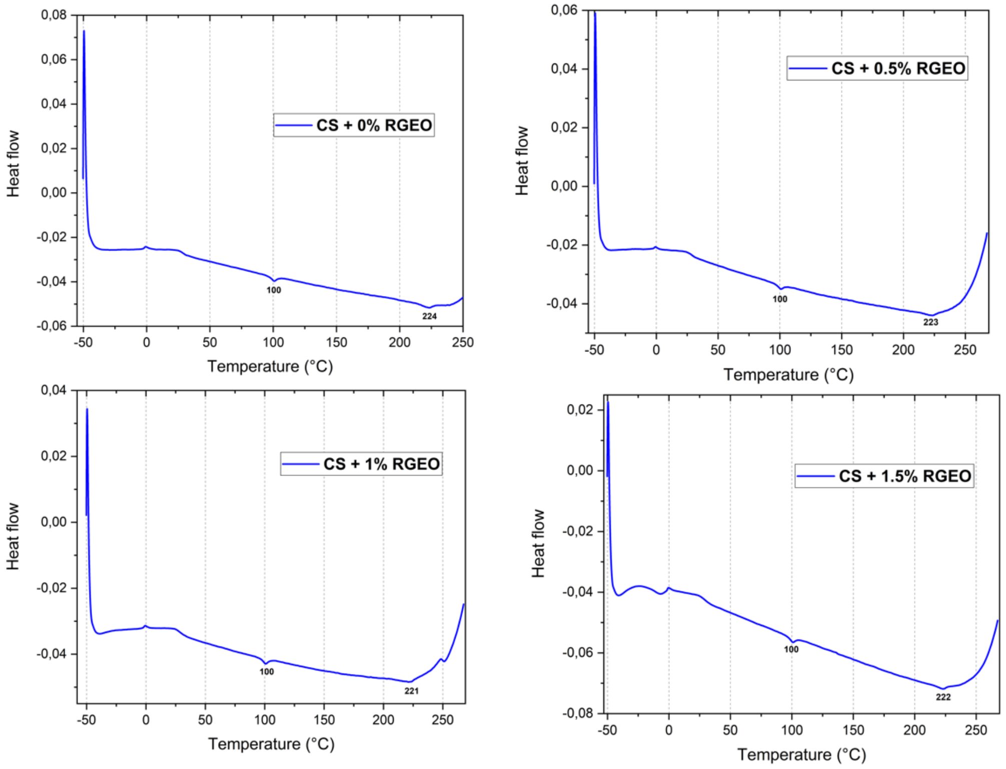

Figure S3. Differential scanning calorimetry analysis (DSC) of CS+RGEO films.
